# Genetic and environmental factors strongly influence risk, severity and progression of age-related macular degeneration

**DOI:** 10.1038/sigtrans.2016.16

**Published:** 2016-09-16

**Authors:** Wenqiu Wang, Katarzyna Gawlik, Joe Lopez, Cindy Wen, Jie Zhu, Frances Wu, William Shi, Samuel Scheibler, Huimin Cai, Ram Vairavan, Alexander Shi, Weldon Haw, Henry Ferreyra, Ming Zhang, Sherman Chang, Kang Zhang

**Affiliations:** 1 Shiley Eye Institute and Institute for Genomic Medicine, San Diego, CA, USA; 2 AutoGenomics Inc., Vista, CA, USA; 3 Department of Ophthalmology and Molecular Medicine Research Center, West China Hospital, Sichuan University, Sichuan, China; 4 Guangzhou KangRui Biological Pharmaceutical Technology Company, Guangzhou, China; 5 Veterans Administration Healthcare System, San Diego, CA, USA

## Abstract

Age-related macular degeneration (AMD) is characterized by complex interactions between genetic and environmental factors. Here we genotyped the selected 25 single-nucleotide polymorphisms (SNPs) in 983 cases with advanced AMD and 271 cases with intermediate AMD and build an AMD life-risk score model for assessment of progression from intermediate to advanced AMD. We analyzed the performance of the prediction model for geographic atrophy progressors or choroidal neovascularization progressors versus non-progressors based on the 25 SNPs plus body mass index and smoking status. Our results suggest that a class prediction algorithm can be used for the risk assessment of progression from intermediate to late AMD stages. The algorithm could also be potentially applied for therapeutic response, and toward personalized care and precision medicine.

## Introduction

Age-related macular degeneration, an ocular degenerative disease, is regarded as the leading cause for irreversible vision loss in people age 55 or older in developed countries.^1^ The typical clinical signs of early AMD are drusen and retinal pigmental epithelium changes. The natural history of AMD is progressive, with gradual loss of visual function that may span over many years’ time. In 10–15% of patients with dry AMD, the deterioration is more rapid and extensive and they suffer significant vision loss due to geographic atrophy (GA). In another approximately 10–15% of patients, the condition progresses to the ‘wet’ or neovascular form (also known as choroidal neovascularization, CNV). Approximately eight million people in the United States suffer symptoms of early or intermediate AMD, of whom almost one million will develop late AMD within the next 5 years. As aging population booms globally, AMD appears to be a significant public concern for the health care departments and scientific researchers.^2,3^

AMD, a heterogeneous and genetically complex disease, is triggered by multiple environmental and genetic risk factors.^4,5^ Epidemiological studies provided solid evidence that environmental factors including BMI (body mass index) and smoking increased risks of AMD. Our previous published study, as wells as other studies (such as AREDS studies),^6–10^ showed that several genetic variants linked strong association with AMD such as CFH, CFB, HTRA1/ARMS2 and others. A combined risk score including these multiple genetic loci along with demographic and environmental data was highly predictive of AMD phenotype.^11–13^ Although GA and CNV are regarded as late AMD suffering poor visual outcomes, GA and CNV require quite different treatment strategies accordingly due to their specific and different histopathological changes. Unfortunately, none of the reported assessment methods were able to precisely predict progression to the subtype of late AMD.

In the current study, we reported new prediction models for assessment of AMD development and disease progression based on 25 highly associated single-nucleotide polymorphisms (SNPs) from 15 genes (Supplementary Table 1) and 2 epidemiological factors. Each genetic or environmental risk factor was previously evaluated for association with AMD development and/or progression to different AMD stages. The lifetime risk model is capable to calculate the probability of developing AMD during the lifespan of an individual, although risk models for progression are providing the likelihood of progression from intermediate AMD stage to GA and CNV. We also proposed, based on the risk score distribution, the stratification approach that might provide a useful clinical tool.

## Materials and methods

### Study population

The study was approved by the institutional review boards of the University of California, San Diego, United states and West China Hospital, Chengdu, China. Informed written consent was obtained from each patient, and protocols were reviewed and approved by local ethics committees. All research adhered to the tenets of the Declaration of Helsinki. Between 2005 and 2010, patients were enrolled in a prospective study at Shiley Eye Institute and West China Hospital. All patients were examined by two experienced ophthalmologists and divided into three groups: (1) advanced AMD cases with geographic atrophy (anywhere within the grid and without any record of hemorrhage) or choroidal neovascularization (hemorrhagic retinal detachment, hemorrhage under the retina or retinal pigment epithelium, subretinal fibrosis) in the worse eye; (2) intermediate AMD cases with macular drusen (small, hard, large, large confluent, semigranual, cuticular, familial, soft confluent and soft no-confluent) >63 μm in the worse eye; (3) controls without known advanced or intermediate AMD. Smoking history was obtained at the baseline visit from questionnaires and participants were classified as current smokers, past smokers, or never smokers. BMI was derived from height and weight measurements at the baseline visit.

### Genotyping

Genomic DNA was extracted from peripheral blood leukocytes according to established protocols^11^ and genotyped by AutoGenomics using INFINITI High Throughput System (Vista, CA, USA) and AMD Panel (Vista, CA, USA, for research use only). On the INFINITI platform, dual levels of specificity are achieved by the multiplexing PCR and allele-specific primer extension (ASPE) processes. Target regions of relevant genes were amplified and the amplicons were served as templates for the ASPE, during which, fluorescent-labeled nucleotides were incorporated for signal amplification. Subsequently, ASPE primers were captured via hybridization of the primer's Tag-region with the anti-Tag oligonucleotides addressed on the BioFilmChip (Vista, CA, USA). The microarray was washed, dried and scanned for the data analysis using the INFINITI ACE Reader (Vista, CA, USA).

### Model building and internal validation

Class prediction model building and testing were performed using TreeNet software (Salford Systems, San Diego, CA, USA). The binary logistic regression analysis with a 10-fold cross-validation method was applied in the algorithm development. TreeNet (https://www.salford-systems.com/products/TreeNet) randomly assigned 9/10th of the data (learning set) to the model building to compute the regression equation and thus establish the risk score model. Remaining 1/10th of the data were used for testing and served as the internal control. Receiver operating characteristic curve (ROC) was computed for both, the learning and testing sets. The closer ROC is between the learning and testing sets, the better class prediction algorithm is developed.

### Statistical analyses

Statistical analyses were performed using SPSS 22.0 (SPSS, Chicago, IL, USA). All values are reported as the mean±s.d. or 95% confidence interval. *P*-values<0.05 were considered statistically significant.

## Results

### Patient information

A total of 1677 unrelated individuals (957 Caucasians, 647 Asins, 39 Hispanics and 34 African Americans) were involved in this 7-year study including 983 patients with advanced AMD (269 diagnosed with geographic atrophy and 714 with choroidal neovascularization), 271 patients with intermediate AMD, and 423 control patients. Patients with intermediate AMD were followed up for 7 years and did not progress to advanced stages, thus were considered non-progressors. Patients diagnosed with late AMD (geographic atrophy or choroidal neovascularization) were considered progressors. Table 1 shows the baseline characteristics of the study participants.

### SNPs selection and genotyping

The following 15 loci with 25 common SNPs and established association with AMD were included in the present study to develop class prediction algorithms for lifetime risk assessment and disease progression: *ABCA1* (rs1883025); *APOE* (rs429358, rs7412); *ARMS2* (rs10490924); *C3* (rs2230199); *CCDC109B* (rs17440077); *CETP* (rs3764261); *CFB* (rs4151669, rs522162); *CFH* (rs1048663, rs1061170, rs10737680, rs1329428, rs2274700, rs3766405, rs412852); *CFI* (rs10033900); *COL8A1* (rs13095226); *HTRA1* (rs11200638); *LIPC* (rs493258, rs10468017); *LPL* (rs12678919); *TIMP3* (rs9621532) and *VEGFA* (rs3025000, rs943080). A summary of the selected SNPs with the risk and non-risk alleles according to the literature, their affected genes and proposed roles in AMD pathogenesis is displayed in Supplementary Table 1.

We genotyped the selected 25 SNPs in 983 cases with advanced AMD, 271 cases with intermediate AMD, and 423 controls. We implemented stringent quality control criteria for each SNP in our data set. All variants showed high genotyping quality with an average call rate >99.5%.

### Risk prediction for developing AMD

**Figure 1:**
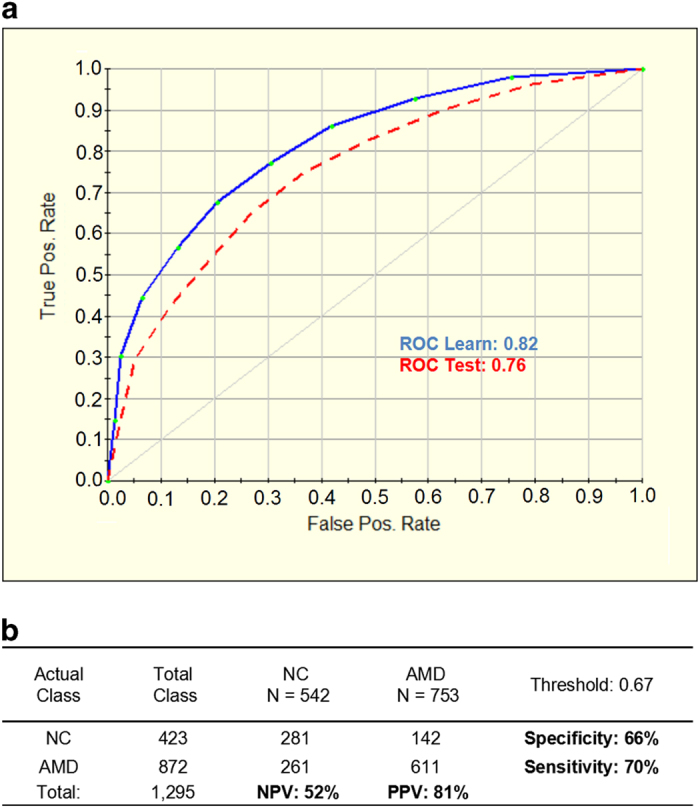
Performance of the model for AMD lifetime risk assessment based on 25 variables (genetic factors only). (**a**) Receiver operating characteristic curve (ROC) for the 25-SNP model was generated for learning (blue line) and testing (red dashed line) sets by using the binary logistic regression analysis with a 10-fold cross-validation method. (**b**) Prediction success parameters were calculated for testing set. AMD, age-related macular degeneration cases; NC, normal control; NPV, negative prediction value; PPV, positive prediction value.

We first assessed AMD lifetime risk defined as the likelihood of developing the disease during the individual’s lifespan based only on genetic factors in our case–control study. We analyzed the performance of the prediction model in AMD patients (872 cases consisting of 269 GA and 603 CNV) versus controls (423 cases) based on the 25 SNPs shown to be associated with the disease. The receiver operating characteristic curve (ROC) for the test set was 0.76 with 70% sensitivity and 66% specificity, where the positive (PPV) and negative (NPV) prediction values were 81% and 52%, respectively (Figure 1).

**Figure 2:**
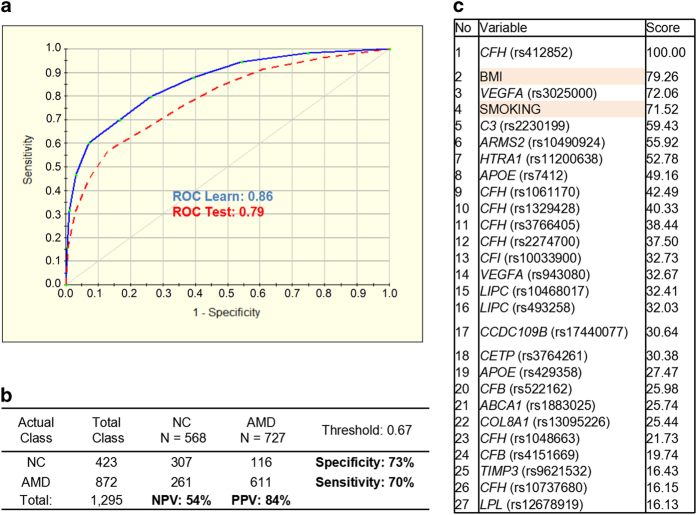
Performance of the model for AMD lifetime risk assessment based on 27 variables (genetic and environmental factors). (**a**) Receiver operating characteristic curve (ROC) for the 25-SNP plus smoking and BMI (body mass index) model was generated for learning (blue line) and testing (red dashed line) sets by using the binary logistic regression analysis with a 10-fold cross-validation method. (**b**) Prediction success parameters were calculated for testing set. (**c**) Variable importance ranking is showing relative scores generated by TreeNet software and positioning the predictors from the most important to the least important. AMD, age-related macular degeneration cases; NC, normal control; NPV, negative prediction value; PPV, positive prediction value.

**Figure 3:**
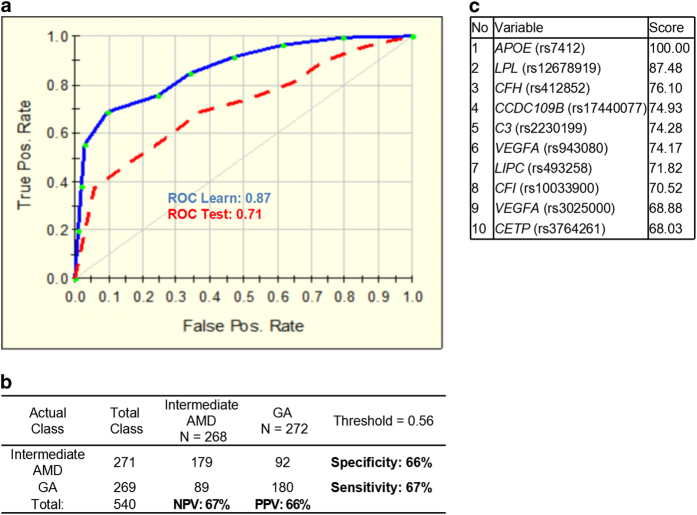
Distribution of the risk score computed in the prediction models. (**a**) AMD lifetime risk score distribution in the study population. AMD cases are shown in red and controls in blue. (**b**) GA progression risk score distribution in the study population. GA progressors are shown in brown and non-progressors in green. (**c**) CNV progression risk score distribution in the study population. CNV progressors are shown in purple and non-progressors in green.

To improve the performance of the model, we added non-genetic modifiable risk factors, BMI and smoking history (current, past or never). For this 27 variable model, the ROC for the test set was 0.79 with 70% sensitivity and 73% specificity, where PPV and NPV values were 84% and 54%, respectively (Figures 2a and b). The variable ranking showed the most important SNPs associated with AMD, along with non-genetic risk factors, at the top including *CFH* (rs412852), *VEGFA* (rs3025000), *C3* (rs2230199) and *ARMS2* (rs10490924), which is in accordance with previously published data (Figure 2c). The AMD lifetime risk prediction model developed based on the 27 predictors (both genetic and non-genetic factors) was shown to be the most effective. The distribution of AMD lifetime risk score for cases and controls as observed in our study is given in Figure 3a. On the basis of the above results, the relative risk score of developing AMD can be generated and grouped into three risk categories: (1) low relative risk of <35%; (2) moderate relative risk of 35–67%; and (3) high relative risk of >67%. These results suggest that a class prediction algorithm can be used for the risk assessment of developing AMD successfully.

### Risk prediction for progression to geographic atrophy and choroidal neovascularization

**Figure 4:**
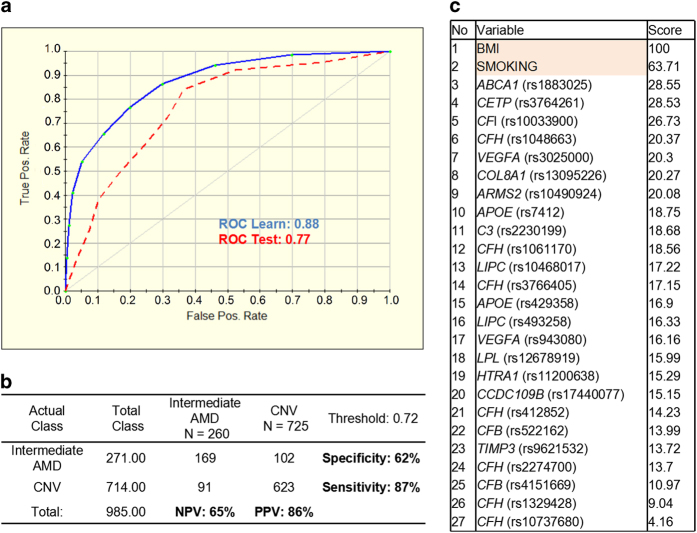
Performance of the prediction model for 7-year progression to geographic atrophy (GA) based on 10 genetic variables. (**a**) Receiver operating characteristic curve (ROC) for the 10-SNP model was generated for learning (blue line) and testing (red dashed line) sets by using the binary logistic regression analysis with a 10-fold cross-validation method. (**b**) Prediction success parameters were calculated for testing set. (**c**) Variable importance ranking is showing relative scores generated by TreeNet software and positioning the predictors from the most important to the least important. NPV, negative prediction value; PPV, positive prediction value.

We hypothesized that when applied to a group of patients with well-characterized intermediate AMD phenotype, a class prediction algorithm might also be used to predict disease progression. Progression was defined as transition from intermediate AMD to advanced AMD, either geographic atrophy (GA) or choroidal neovascularization (CNV), in the worse eye during a follow-up visit. We selected only patients with intermediate AMD who did not progress within 7 years to advanced AMD and considered them as non-progressors. Here we analyzed the performance of the prediction model for GA progressors (269 cases) or CNV progressors (714 cases) versus non-progressors (271 cases with intermediate AMD) based on the 27 variables (25 SNPs, BMI and smoking status). For both approaches we applied a ‘shaving’ technique, in which the predictors are ranked from top to bottom (the most important to the least important) at every step when the bottom predictor is removed and the model is rebuilt. This technique allowed us to choose the model for risk of progression to GA based on 10 variables with the best performance (Figure 4). The ROC for the test set was 0.71 with 67% sensitivity and 66% specificity, where PPV and NPV values were 66% and 67%, respectively (Figures 4a and b). The variable ranking (Figure 4c) showed only genetic factors at the top including *APOE* (rs7412), *LPL* (rs12678919), *CFH* (rs412852) and *CCDC109B* (rs17440077).

**Figure 5:**
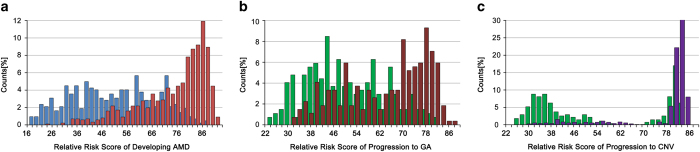
Performance of the prediction model for 7-year progression to choroidal neovascularization (CNV) based on 27 variables (genetic and environmental factors). (**a**) Receiver operating characteristic curve (ROC) for the 25-SNP plus smoking and BMI model was generated for learning (blue line) and testing (red dashed line) sets by using the binary logistic regression analysis with a 10-fold cross-validation method. (**b**) Prediction success parameters were calculated for testing set. (**c**) Variable importance ranking is showing relative scores generated by TreeNet software and positioning the predictors from the most important to the least important. NPV, negative prediction value; PPV, positive prediction value.

Using a ‘shaving’ technique for the modeling of progression risk to CNV, we were not able to narrow down the number of the predictors and find an algorithm with better performance. Thus we evaluated the prediction model for risk of progression to CNV based on 27 variables (genetic and environmental factors). The ROC for the test set was 0.77 with 87% sensitivity and 62% specificity, where PPV and NPV values were 86% and 65%, respectively (Figures 5a and b). The variable ranking (Figure 5c) showed non-genetic risk factors at the top along with SNPs including *ABCA1* (rs1883025), *CETP* (rs3764261), *CFI* (rs10033900) and *CFH* (rs1048663). The distribution of GA and CNV progression risk score for progressors and non-progressors as observed in our study is given in Figures 3b and c. Our results suggest that a class prediction algorithm can also be used for the risk assessment of progression from intermediate to late AMD stages.

## Discussion

In the current study, we tested a panel of 25 SNPs for AMD association in 983 unrelated individuals diagnosed with late AMD, 271 patients with intermediate AMD and 423 controls. We demonstrated that multiple SNPs were sufficient to assess the AMD lifetime risk. Along with the non-genetic factors, smoking status and BMI, the algorithm was shown to be highly predictive and could differentiate between low, medium and high risk of developing AMD.

Before the first class prediction algorithm for AMD risk assessment was developed, we tested if a class prediction algorithm can be applied to categorical variables such as genotyping data. We found that a multivariate approach from the class prediction algorithm allowed the descriptive genotype data to be used directly for AMD risk prediction model building with comparable performance to the model built based on the numeric variables, thus eliminating the necessity of odds ratios calculations (Supplementary Figure 1).

AMD lifetime risk score is specifically developed to tell the likelihood of developing AMD during the lifespan of an individual. At first, we assessed our cases and controls based only on 25 genetic factors and the ROC was 0.76 with 70% sensitivity and 66% specificity. After adjusting for the smoking status and BMI, the actual lifetime risk ROC increased to 0.79 with 70% sensitivity and 73% specificity, which indicated that smoking and BMI were involved as important AMD risk factors. According to our lifetime risk model, the relative score values of 8 variables: *CFH* (rs412852), BMI, *VEGFA* (rs3025000), SMOKING, *C3* (rs2230199), *ARMS2* (rs10490924), *HTRA1* (rs11200638) and *APOE* (rs7412) were close to or >50, what proved their high contribution to the AMD development and reliability of the prediction model. Although there are no lifetime risk models reported elsewhere based exactly on the same combination of risk factors that were used here, they have been identified to have strong association with increased risk of AMD.^11,14–23^ The use of individual lifetime risk prediction might help to increased awareness of, and interest in, the importance and prevention of AMD in large population. They can also be used to guide the allocation of resources to improve public health services for AMD from both clinicians and the general public.

AMD is a slowly progressive retinal disease, and its course is variable and individual. In the current study, we defined progression as transition from intermediate AMD to advanced AMD, either geographic atrophy (GA) or choroidal neovascularization (CNV), in the worse eye during a follow-up visit. To identify whether association results differ between two subtypes of late AMD, we built two progression prediction models, respectively. In GA prediction model, 10 variables had been found to obtain the best performance and further evaluate with 67% sensitivity and 66% specificity. CNV prediction model was established based on 27 variables with 87% sensitivity and 62% specificity. Contrary to the CNV model, the GA model comprised none of the non-genetic factors, which suggests that genetic factors might have a predominant role in progression to GA.

Surprisingly, in CNV model, BMI and smoking status showed larger contribution to CNV progression than genetic factors. BMI and smoking are the most important modifiable environmental risk factors associated with AMD. Obesity was shown to be associated with the incidence of AMD (OR=1.04 per kg m^−2^) in age, gene/environment susceptibility (AGES) study. Seddon, George *et al.*^24^ found that BMI of 25 kg m^−2^ or higher was found to increase the risk of advanced AMD for CT heterozygotes in CFH Y402H. For smoking, the risk of developing AMD was 3.7-fold higher in current smokers, and 1.8-fold higher in past smokers when compared with patients who never smoked.^11,25^ However, considered as self-estimated factors, weight control and smoking quitting were usually avoided to be assessed in most studies for CNV treatments. Although the way how the non-genetic factors acted on CNV course keeps unclear, our results provide an evidence for healthy lifestyle in preventing or slow-downing the progression of CNV.

Four SNPs, *CFI* (rs10033900), *CETP* (rs3764261), *VEGFA* (rs3025000) and *APOE* (rs7412), are found to overlap in top 10 variables of GA and CNV prediction. It may not necessarily reflect a shared etiology, but it may not necessarily reflect a shared etiology but may represent overlapping risk factors common to GA and CNV. On the other hand, it cannot be excluded that GA and CNV share a common etiology with slightly different end-stage manifestation. In spite of these encouraging prediction results of individuals with CNV and GA, additional specific gene–gene and gene–environment interactions are still required to confirm these findings and to complement the relative weakness of the GA prediction.

The limitations of the presented models include the restriction of our analyses to mostly Caucasian population. The same polymorphism plays a different role in different ethnic populations or across different studies. The sample size for AMD risk prediction model is not large. We still need to be careful when extending the results to other populations. Nevertheless, we successfully built a prediction algorithm for risk of developing AMD and disease progression, and provided a strong basis for conducting future studies with much larger sample size to validate the initial findings.

## Figures and Tables

**Table 1:** Summary of demographic characteristics for study participants

	*Control*	*AMD*		
	n*=423*	*CNV (*n*=714)*	*GA (*n*=269)*	*Intermediate (*n*=271)*
*Age*				
media (range)	68 (49, 96)	77 (44, 97)	81 (49,101)	82 (47,107)
				
*BMI*				
Mean±s.d.	26.70±5.53	26.47±5.21	26.66±5.34	26.02±5.16
				
*Sex (*n)				
Female	223 (52.7%)	455 (63.7%)	161 (59.8%)	155 (57.2%)
Male	200 (47.3%)	259 (36.3%)	108 (40.2%)	116 (42.8%)
				
*Smoking (*n)				
Past	143 (33.8%)	318 (44.5%)	79 (29.4%)	75 (27.7%)
Never	256 (60.5%)	282 (39.5%)	102 (37.9%)	141 (52.0%)
Current	17 (4.0%)	42 (5.9%)	8 (3.0%)	5 (1.8%)
NA	7 (1.7%)	72 (10.1%)	80 (29.7%)	50 (18.5%)

Abbreviations: AMD, age-related macular degeneration; BMI, body mass index; CNV, choroidal neovascularization; GA, geographic atrophy; NA, data not available.
