# Supplementary material for: Genetic and environmental factors strongly influence risk, severity and progression of age-related macular degeneration
Source: Signal Transduct Target Ther. 2016 Sep 16;1:16016–. doi: 10.1038/sigtrans.2016.16 (PMC5661646; doi:10.1038/sigtrans.2016.16)
Supplement: Supplementary Information [file sigtrans201616-s1.doc]

**Supplementary information**
**Supplementary Table 1. Summary information of 25 SNPs associated with AMD**

**Supplementary Figure 1.** Performance of the AMD prediction algorithms built based on categorical (genotype data) and numerical (odds ratios) variables. Receiver operating characteristic curve (ROC) for the model built on genotype data (**a**) and for the model built based on odds ratios (**c**) was generated for learning (blue line) and testing (red dashed line) sets by using the binary logistic regression analysis with a 10-fold cross-validation method. Prediction success parameters for the model built on genotype data (**c**) and for the model built based on odds ratios (**d**) were calculated for testing set.
