# Supplementary material for: Genetic and environmental factors strongly influence risk, severity and progression of age-related macular degeneration
Source: Signal Transduct Target Ther. 2016 Sep 16;1:16016–. doi: 10.1038/sigtrans.2016.16 (PMC5661646; doi:10.1038/sigtrans.2016.16)
Supplement: Supplementary Table 1 [file sigtrans201616-s2.doc]

**Supplementary**

**Table1. Summary information of 25 Single nucleotide polymorphisms (SNPs) associated with AMD**

| **No.** | **SNP** | **Gene** | **Pathways/Functions** |  | **Risk Allele** | **Non-Risk Allele** |  | **References** |
| --- | --- | --- | --- | --- | --- | --- | --- | --- |
| 1 | rs3766405 | ***CFH*** | Complement |  | C | T | primarily western European ancestry | Li M, et al.^1^ |
| 2 | rs412852 | ***CFH*** |  |  | C | T |  |  |
| 3 | rs1048663 | ***CFH*** |  |  | G | A |  |  |
| 4 | rs1061170 | ***CFH*** |  |  | C | T | predominantly Caucasian | Bergeron-Sawitzke J, et al.^2^ |
| 5 | rs1329428 | ***CFH*** |  |  | G | A |  | Li M, et al.^1^ |
| 6 | rs2274700 | ***CFH*** |  |  | C | T | predominantly Caucasian | Chen Y, et al. ^3^ |
| 7 | rs10737680 | ***CFH*** |  |  | A | C |  | Li M, et al.^1^ |
| 8 | rs2230199 | ***C3*** | Complement |  | G | C | predominantly of European ancestry, the Han Chinese in Beijing, China and Japanese in Tokyo, Japan | Fritsche LG, et al. ^4^ |
| 9 | rs10033900 | ***CFI*** | Complement |  | T | C | Caucasian individuals | Reynolds R, et al. ^5^ |
| 10 | rs9621532 | ***TIMP3*** | Extracellular matrix degradation |  | A | C | JHU, COL, Genentech, Iceland, Wash-U, AUS, RS, FR-CRET and Irish | Yu Y, et al.^6^ |
| 11 | rs12678919 | ***LPL*** | Lipoprotein metabolism |  | G | A | NA | Chen W, et al.^7^ |
| 12 | rs493258 | ***LIPC*** | Lipoprotein metabolism, atherosclerosis |  | G | A | Caucasian individuals | Lee J, et al.^8^ |
| 13 | rs10468017 | ***LIPC*** |  |  | C | T |  |  |
| 14 | rs1883025 | ***ABCA1*** | Cellular lipid removal |  | G | A |  | Chen W, et al.^7^ |
| 15 | rs10490924 | ***ARMS2*** | Uncertain, possibly mitochondrial |  | T | G |  | Bergeron-Sawitzke J, et al.^2^; Fritsche LG, et al. ^4^ |
| 16 | rs11200638 | ***HTRA1*** | Cell growth |  | A | G |  | Bergeron-Sawitzke J, et al.^2^ |
| 17 | rs3764261 | ***CETP*** | Lipoprotein metabolism, atherosclerosis |  | A | C |  | Chen W, et al.^7^ |
| 18 | rs429358 | ***APOE*** | Lipoprotein metabolism, atherosclerosis |  | T | C | Australia，who are descendants of Northern Europeans | Adams MK, et al. ^9^ |
| 19 | rs7412 | ***APOE*** |  |  | C | T |  |  |
| 20 | rs4151669 | ***CFB*** | Complement |  | A | G | India | Kaur I, et al. ^10^ |
| 21 | rs522162 | ***CFB*** |  |  | G | A | Caucasian | Naj AC, et al. ^11^ |
| 22 | rs13095226 | ***COL8A1*** | Extracellular matrix |  | C | T | European ancestry | Neale BM, et al.^12^ |
| 23 | rs3025000 | ***VEGFA*** | Angiogenesis |  | T | C | Australia | Abedi F, et al. ^13^ |
| 24 | rs943080 | ***VEGFA*** |  |  | T | C | European and Asian ancestry | Zhao L, et al. ^14^ ; Fritsche LG, et al.^15^ |
| 25 | rs17440077 | ***CCDC109B*** | Uncertain |  | G | A | predominantly Caucasian | Yaspan BL, et al. ^16^ |

**Reference**

1. Li M, Atmaca-Sonmez P, Othman M, et al. CFH haplotypes without the Y402H coding variant show strong association with susceptibility to age-related macular degeneration. *Nature genetics.* 2006;38(9):1049-1054.
2. Bergeron-Sawitzke J, Gold B, Olsh A, et al. Multilocus analysis of age-related macular degeneration. *European journal of human genetics : EJHG.* 2009;17(9):1190-1199.
3. Chen Y, Zeng J, Zhao C, et al. Assessing susceptibility to age-related macular degeneration with genetic markers and environmental factors. *Archives of ophthalmology.* 2011;129(3):344-351.
4. Fritsche LG, Igl W, Bailey JN, et al. A large genome-wide association study of age-related macular degeneration highlights contributions of rare and common variants. *Nature genetics.* 2016;48(2):134-143.
5. Reynolds R, Hartnett ME, Atkinson JP, Giclas PC, Rosner B, Seddon JM. Plasma complement components and activation fragments: associations with age-related macular degeneration genotypes and phenotypes. *Investigative ophthalmology & visual science.* 2009;50(12):5818-5827.
6. Yu Y, Bhangale TR, Fagerness J, et al. Common variants near FRK/COL10A1 and VEGFA are associated with advanced age-related macular degeneration. *Human molecular genetics.* 2011;20(18):3699-3709.
7. Chen W, Stambolian D, Edwards AO, et al. Genetic variants near TIMP3 and high-density lipoprotein-associated loci influence susceptibility to age-related macular degeneration. *Proceedings of the National Academy of Sciences of the United States of America.* 2010;107(16):7401-7406.
8. Lee J, Zeng J, Hughes G, et al. Association of LIPC and advanced age-related macular degeneration. *Eye.* 2013;27(2):265-270; quiz 271.
9. Adams MK, Simpson JA, Richardson AJ, et al. Apolipoprotein E gene associations in age-related macular degeneration: the Melbourne Collaborative Cohort Study. *American journal of epidemiology.* 2012;175(6):511-518.
10. Kaur I, Katta S, Reddy RK, et al. The involvement of complement factor B and complement component C2 in an Indian cohort with age-related macular degeneration. *Investigative ophthalmology & visual science.* 2010;51(1):59-63.
11. Naj AC, Scott WK, Courtenay MD, et al. Genetic factors in nonsmokers with age-related macular degeneration revealed through genome-wide gene-environment interaction analysis. *Annals of human genetics.* 2013;77(3):215-231.
12. Neale BM, Fagerness J, Reynolds R, et al. Genome-wide association study of advanced age-related macular degeneration identifies a role of the hepatic lipase gene (LIPC). *Proceedings of the National Academy of Sciences of the United States of America.* 2010;107(16):7395-7400.
13. Abedi F, Wickremasinghe S, Richardson AJ, et al. Variants in the VEGFA gene and treatment outcome after anti-VEGF treatment for neovascular age-related macular degeneration. *Ophthalmology.* 2013;120(1):115-121.
14. Zhao L, Grob S, Avery R, et al. Common variant in VEGFA and response to anti-VEGF therapy for neovascular age-related macular degeneration. *Current molecular medicine.* 2013;13(6):929-934.
15. Fritsche LG, Chen W, Schu M, et al. Seven new loci associated with age-related macular degeneration. *Nature genetics.* 2013;45(4):433-439, 439e431-432.
16. Yaspan B, Li Z; Dressen A, et al. A Common SNP at the CFI Locus is Associated with Rapid Progression of Geographic Atrophy. *Investigative Ophthalmology & Visual Science,* 2014;55(4), 2234.
