# Supplementary figures and images for: Genetic and environmental factors strongly influence risk, severity and progression of age-related macular degeneration
Source: Signal Transduct Target Ther. 2016 Sep 16;1:16016–. doi: 10.1038/sigtrans.2016.16 (PMC5661646; doi:10.1038/sigtrans.2016.16)

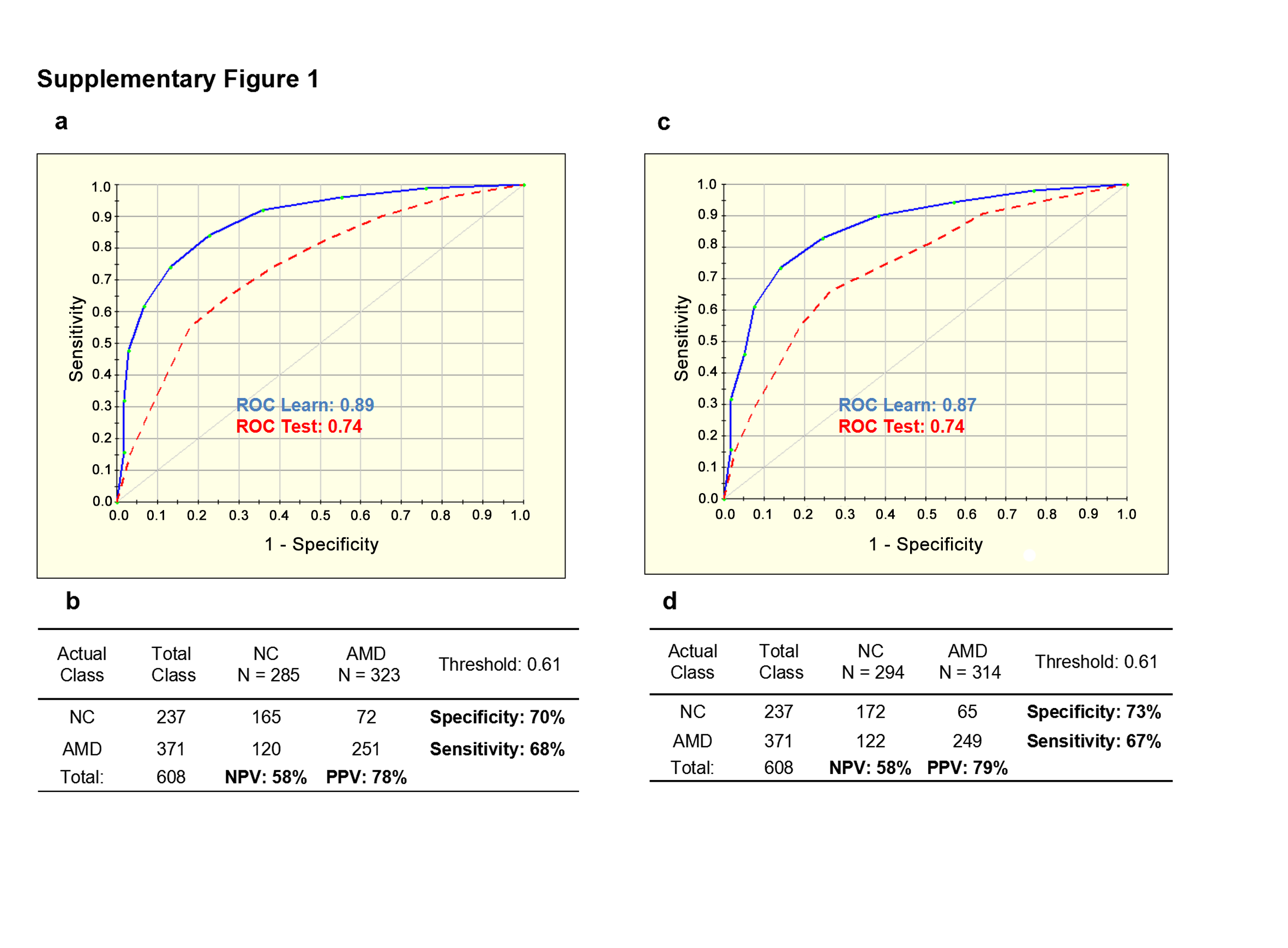

Supplement: Supplementary Figure 1 [file sigtrans201616-s3.tiff]
